# Supplementary material for: Electronic excited states in deep variational Monte Carlo
Source: Nat Commun. 2023 Jan 17;14:274. doi: 10.1038/s41467-022-35534-5 (PMC9845370; doi:10.1038/s41467-022-35534-5)
Supplement: Supplementary file 1 — Supplementary Information for Electronic excited states in deep variational Monte Carlo [file 41467_2022_35534_MOESM1_ESM.pdf]

# Supplementary Information

# Electronic excited states in deep variational Monte Carlo

M. T. Entwistle,[1,*] Z. Schätzle,[1,*] P. A. Erdman,[1] J. Hermann,[1,†] and F. Noé[2,1,3,4,‡]

[1] *FU Berlin, Department of Mathematics and Computer Science, Arnimallee 12, 14195 Berlin, Germany*
[2] *Microsoft Research AI4Science*
[3] *FU Berlin, Department of Physics, Arnimallee 14, 14195 Berlin, Germany*
[4] *Rice University, Department of Chemistry, Houston, Texas 77005, USA*

This document provides details on the test systems and our calculations as detailed in the main paper.

## SUPPLEMENTARY NOTE I: GEOMETRIES OF TEST SYSTEMS

All calculations presented in Sections II A – C of the main paper were done at the ground-state equilibrium geometries. The geometries for all molecules are listed in Supplementary Table I. For the ethylene structures presented in Section II D, we list the planar (ground-state equilibrium) and twisted ($\tau = 90$) geometries.

Supplementary Table I: Ground-state equilibrium geometries of the test systems.

| Molecule | Atom | Position (Å) | Molecule | Atom | Position (Å) |
|---|---|---|---|---|---|
| LiH | Li | (0.000, 0.000, 0.000) | BeH | Be | (0.000000, 0.000000, 0.000000) |
| | H | (1.595, 0.000, 0.000) | | H | (1.326903, 0.000000, 0.000000) |
| BH | B | (0.000000, 0.000000, 0.000000) | $CH^+$ | C | (0.00000, 0.00000, 0.00000) |
| | H | (0.000000, 0.000000, 1.222874) | | H | (1.13092, 0.00000, 0.00000) |
| $H_2O$ | O | (0.000000, 0.000000, -0.069903) | CO | C | (0.000000, 0.000000, -0.661165) |
| | H | (0.000000, 0.757532, 0.518435) | | O | (0.000000, 0.000000, 0.472379) |
| | H | (0.000000, -0.757532, 0.518435) | | | |
| $NH_3$ | N | (0.067759, -0.000000, 0.000000) | $C_2H_4$ (planar) | C | (-0.675000, 0.000000, 0.000000) |
| | H | (-0.313823, 0.468746, -0.811891) | | C | (0.675000, 0.000000, 0.000000) |
| | H | (-0.313823, -0.937491, -0.000000) | | H | (-1.242900, 0.000000, -0.930370) |
| | H | (-0.313823, 0.468746, 0.811891) | | H | (-1.242900, 0.000000, 0.930370) |
| | | | | H | (1.242900, 0.000000, -0.930370) |
| | | | | H | (1.242900, 0.000000, 0.930370) |
| $C_2H_4$ (twisted) | C | (-0.688500, 0.000000, 0.000000) | $C_6H_6$ | C | (0.000000, 1.396792, 0.000000) |
| | C | (0.688500, 0.000000, 0.000000) | | C | (0.000000, -1.396792, 0.000000) |
| | H | (-1.307207, 0.000000, -0.915547) | | C | (1.209657, 0.698396, 0.000000) |
| | H | (-1.307207, 0.000000, 0.915547) | | C | (-1.209657, -0.698396, 0.000000) |
| | H | (1.307207, -0.915547, 0.000000) | | C | (-1.209657, 0.698396, 0.000000) |
| | H | (1.307207, 0.915547, 0.000000) | | C | (1.209657, -0.698396, 0.000000) |
| | | | | H | (0.000000, 2.484212, 0.000000) |
| | | | | H | (2.151390, 1.242106, 0.000000) |
| | | | | H | (-2.151390, -1.242106, 0.000000) |
| | | | | H | (-2.151390, 1.242106, 0.000000) |
| | | | | H | (2.151390, -1.242106, 0.000000) |
| | | | | H | (0.000000, -2.484212, 0.000000) |

* M. T. Entwistle and Z. Schätzle contributed equally to this work.
† science@jan.hermann.name
‡ franknoe@microsoft.com

## SUPPLEMENTARY NOTE II: ENERGIES FOR SMALL ATOMS AND MOLECULES

We tabulate the total energies and vertical excitation energies that were obtained for the small systems, as presented in Section II A of the main paper. These are listed in Supplementary Table II.

Supplementary Table II: Total energies and vertical excitation energies (both in Ha) for the small atoms in Section II A.

| System | $E_n$ | Correlation energy (%) | $\Delta E$ | $\Delta E$ ($\sigma^2$ matching) |
|---|---|---|---|---|
| Li | -7.47800(4) | 99.9(1)[a] | — | — |
| | -7.40998(5) | — | 0.06801(7) | 0.06807(7) |
| | -7.40988(7) | — | 0.06812(8) | 0.06811(9) |
| | -7.40977(7) | — | 0.06823(8) | 0.06824(9) |
| | -7.335(4) | — | 0.143(4) | 0.143(4) |
| Be | -14.66736(7) | 100.0(1)[a] | — | — |
| | -14.5663(6) | — | 0.1011(6) | 0.1009(6) |
| | -14.5664(1) | — | 0.1010(1) | 0.1007(2) |
| | -14.5664(1) | — | 0.1010(1) | 0.1004(2) |
| | -14.4710(2) | — | 0.1964(2) | 0.1952(2) |
| B | -24.6509(2) | 97.6(1)[a] | — | — |
| | -24.6506(2) | — | 0.0004(3) | 0.0005(3) |
| | -24.6498(2) | — | 0.0011(3) | 0.0008(3) |
| | -24.5206(2) | — | 0.1303(2) | 0.1300(3) |
| | -24.5202(2) | — | 0.1307(3) | 0.1300(3) |
| C | -37.8388(3) | 96.0(2)[a] | — | — |
| | -37.8388(2) | — | -0.0001(4) | 0.0001(4) |
| | -37.8378(2) | — | 0.0010(4) | 0.0008(4) |
| | -37.7918(2) | — | 0.0470(4) | 0.0470(4) |
| | -37.7911(3) | — | 0.0477(4) | 0.0451(4) |
| N | -54.5836(3) | 97.0(2)[a] | — | — |
| | -54.4911(4) | — | 0.0925(5) | 0.0900(5) |
| | -54.4885(4) | — | 0.0952(5) | 0.0893(6) |
| | -54.4874(4) | — | 0.0963(6) | 0.0883(6) |
| | -54.4861(4) | — | 0.0975(5) | 0.0918(6) |
| O | -75.0532(5) | 94.6(2)[a] | — | — |
| | -75.0506(5) | — | 0.0027(7) | -0.0003(8) |
| | -75.0519(5) | — | 0.0013(7) | -0.0011(7) |
| | -74.9763(6) | — | 0.0769(8) | 0.0711(8) |
| | -74.9771(6) | — | 0.0761(8) | 0.0703(9) |

[a] Exact energy and HF energy at the CBS limit [1]

Supplementary Table II (continued): Total energies and vertical excitation energies (both in Ha) for the many-state calculations in Section II A.

| System | $E_n$ | Correlation energy (%) | $\Delta E$ | $\Delta E$ ($\sigma^2$ matching) |
|---|---|---|---|---|
| LiH | -8.0695(1) | 98.9(1)[a] | — | — |
| | -7.9497(1) | — | 0.1198(1) | 0.1199(1) |
| | -7.9348(1) | — | 0.1348(2) | 0.1347(2) |
| | -7.9112(1) | — | 0.1584(2) | 0.1573(2) |
| | -7.9114(1) | — | 0.1582(1) | 0.1574(2) |
| | -7.8978(1) | — | 0.1717(2) | 0.1710(2) |
| | -7.8977(1) | — | 0.1718(2) | 0.1709(2) |
| | -7.8549(1) | — | 0.2147(2) | 0.2130(2) |
| BeH | -15.2452(4) | 98.3(4)[a] | — | — |
| | -15.1516(2) | — | 0.0936(4) | 0.0916(3) |
| | -15.1507(2) | — | 0.0946(5) | 0.0914(3) |
| | -15.0286(3) | — | 0.2166(5) | 0.2132(4) |
| | -15.0259(2) | — | 0.2193(5) | 0.2156(4) |
| | -15.0249(2) | — | 0.2203(5) | 0.2138(17) |
| | -14.9951(4) | — | 0.2500(5) | 0.2379(5) |
| | -14.9528(3) | — | 0.2923(5) | 0.2793(16) |
| Be | -14.6667(1) | 99.3(1)[a] | — | — |
| | -14.5655(2) | — | 0.1012(2) | 0.1011(2) |
| | -14.5654(1) | — | 0.1013(2) | 0.1007(2) |
| | -14.5651(2) | — | 0.1016(2) | 0.1009(3) |
| | -14.4663(3) | — | 0.2004(4) | 0.1967(10) |
| | -14.4671(3) | — | 0.1996(3) | 0.1976(5) |
| | -14.4665(3) | — | 0.2002(3) | 0.1982(5) |
| | -14.3915(2) | — | 0.2752(2) | 0.2732(4) |

[a] Exact energy and HF energy at the CBS limit [1]

## SUPPLEMENTARY NOTE III: ENERGIES AND OSCILLATOR STRENGTHS FOR INTERMEDIATE SYSTEMS

We tabulate the total energies, vertical excitation energies and oscillator strengths that were obtained for the intermediate systems, as presented in Section II B of the main paper. These are listed in Supplementary Table III.

Supplementary Table III: Total energies (in Ha), vertical excitation energies (in Ha) and oscillator strengths (in au) for the systems in Section II B.

| Molecule | $E_n$ | Correlation energy (%) | $\Delta E$ | $\Delta E$ ($\sigma^2$ matching) | $f$ | $f$ ($\sigma^2$ matching) |
|---|---|---|---|---|---|---|
| BH | -25.2883(2) | 99.5(1)[a] | — | — | — | — |
| | -25.2361(2) | — | 0.0522(3) | 0.0511(3) | — | — |
| | -25.2356(2) | — | 0.0527(3) | 0.0503(3) | — | — |
| | -25.1765(2) | — | 0.1118(3) | 0.1088(3) | 0.0276(1) | 0.0253(1) |
| $CH^+$ | -38.0863(2) | — | — | — | — | — |
| | -38.0385(2) | — | 0.0478(3) | 0.0460(3) | — | — |
| | -38.0375(3) | — | 0.0488(4) | 0.0465(4) | — | — |
| | -37.9664(3) | — | 0.1199(4) | 0.1172(4) | 0.00606(4) | 0.00576(3) |
| $H_2O$ | -76.4230(5) | 95.9(1)[b] | — | — | — | — |
| | -76.1499(6) | — | 0.2731(8) | 0.2702(8) | — | — |
| | -76.1343(6) | — | 0.2887(8) | 0.2845(9) | 0.0395(3) | 0.0374(3) |
| $NH_3$ | -56.5533(3) | 96.6(1)[b] | — | — | — | — |
| | -56.3122(4) | — | 0.2411(5) | 0.2391(6) | — | — |
| | -56.2972(4) | — | 0.2561(5) | 0.2531(6) | 0.0630(4) | 0.0731(5) |
| CO | -113.3039(6) | 95.9(1)[b] | — | — | — | — |
| | -113.0618(7) | — | 0.2421(9) | 0.2386(9) | — | — |
| | -112.9765(7) | — | 0.3274(9) | 0.3211(9) | 0.1048(5) | 0.1030(5) |

[a] Exact energy [2] and HF energy at the CBS limit [3]
[b] Exact energy and HF energy at the CBS limit [1]

## SUPPLEMENTARY NOTE IV: ENERGIES FOR BENZENE

We tabulate the total energies and vertical excitation energies that were obtained for benzene, as presented in Section II C of the main paper. These are listed in Supplementary Table IV.

Supplementary Table IV: Total energies and vertical excitation energies (both in Ha) for benzene in Section II C.

| $E_0$ | $E_1$ | $\Delta E$ | $\Delta E$ ($\sigma^2$ matching) |
|---|---|---|---|
| -232.0675(11) | -231.8628(9) | 0.2047(14) | 0.1637(13) |

## SUPPLEMENTARY NOTE V: ENERGIES FOR ETHYLENE

We tabulate the total energies and vertical excitation energies that were obtained for all ethylene structures, as presented in Section II D of the main paper. These are listed in Supplementary Table V.

Supplementary Table V: Total energies and vertical excitation energies (both in Ha) for ethylene in Section II D.

| $\tau$ ($\phi = 0$) | $E_n$ | $\Delta E$ | $\Delta E$ ($\sigma^2$ matching) | $\phi$ ($\tau = 90°$) | $E_n$ | $\Delta E$ | $\Delta E$ ($\sigma^2$ matching) |
|---|---|---|---|---|---|---|---|
| 0 | -78.5650(4) | — | — | 0 | -78.4477(4) | — | — |
| | -78.2617(5) | 0.3033(6) | 0.2964(7) | | -78.3578(5) | 0.0899(6) | 0.0897(7) |
| 15 | -78.5609(4) | — | — | 20 | -78.4471(4) | — | — |
| | -78.2755(5) | 0.2854(6) | 0.2799(7) | | -78.3589(5) | 0.0882(6) | 0.0869(7) |
| 30 | -78.5461(4) | — | — | 40 | -78.4391(4) | — | — |
| | -78.2925(5) | 0.2536(6) | 0.2459(7) | | -78.3661(5) | 0.0730(6) | 0.0735(6) |
| 45 | -78.5254(4) | — | — | 60 | -78.4267(5) | — | — |
| | -78.3171(5) | 0.2083(6) | 0.2032(7) | | -78.3704(5) | 0.0563(7) | 0.0537(7) |
| 60 | -78.4952(4) | — | — | 70 | -78.4161(5) | — | — |
| | -78.3339(5) | 0.1613(6) | 0.1580(7) | | -78.3721(5) | 0.0440(7) | 0.0428(7) |
| 70 | -78.4771(4) | — | — | 80 | -78.3992(4) | — | — |
| | -78.3416(5) | 0.1355(6) | 0.1324(7) | | -78.3701(5) | 0.0291(6) | 0.0258(7) |
| 80 | -78.4540(5) | — | — | 90 | -78.3828(4) | — | — |
| | -78.3449(5) | 0.1091(7) | 0.1089(7) | | -78.3675(5) | 0.0153(6) | 0.0112(7) |
| 85 | -78.4483(4) | — | — | 95 | -78.3697(4) | — | — |
| | -78.3512(5) | 0.0971(6) | 0.0922(7) | | -78.3612(5) | 0.0085(6) | 0.0083(7) |
| 90 | -78.4425(5) | — | — | 97.5 | -78.3634(5) | — | — |
| | -78.3531(5) | 0.0894(7) | 0.0881(7) | | -78.3593(5) | 0.0041(7) | 0.0023(7) |
| | | | | 100 | -78.3587(5) | — | — |
| | | | | | -78.3559(5) | 0.0028(7) | 0.0014(7) |
| | | | | 102.5 | -78.3552(5) | — | — |
| | | | | | -78.3529(5) | 0.0023(7) | 0.0042(7) |
| | | | | 105 | -78.3509(5) | — | — |
| | | | | | -78.3448(5) | 0.0061(7) | 0.0048(7) |
| | | | | 110 | -78.3449(5) | — | — |
| | | | | | -78.3224(5) | 0.0225(7) | 0.0189(7) |
| | | | | 120 | -78.3284(5) | — | — |
| | | | | | -78.2929(5) | 0.0355(7) | 0.0231(7) |

## SUPPLEMENTARY NOTE VI: OVERLAPS OF TRAINED WAVEFUNCTIONS

For each system we compute the pairwise overlaps between all trained wavefunctions and tabulate the mean. These are listed in Supplementary Table VI.

Supplementary Table VI: Mean pairwise overlaps for all systems.

| System | $\overline{\lvert S_{ij}\rvert}$ | System | $\overline{\lvert S_{ij}\rvert}$ | System | $\overline{\lvert S_{ij}\rvert}$ | System | $\overline{\lvert S_{ij}\rvert}$ |
|---|---|---|---|---|---|---|---|
| Li | 0.0010(6) | $CH^+$ | 0.0018(7) | $C_2H_4$ ($\tau = 80$) | 0.002(2) | $C_2H_4$ ($\phi = 95$) | 0.002(1) |
| Be | 0.004(1) | $H_2O$ | 0.007(3) | $C_2H_4$ ($\tau = 85$) | 0.001(1) | $C_2H_4$ ($\phi = 97.5$) | 0.003(2) |
| B | 0.0023(9) | $NH_3$ | 0.014(3) | $C_2H_4$ ($\tau = 90$) | 0.008(2) | $C_2H_4$ ($\phi = 100$) | 0.002(3) |
| C | 0.0030(4) | CO | 0.003(1) | $C_2H_4$ ($\phi = 0$) | 0.009(1) | $C_2H_4$ ($\phi = 102.5$) | 0.007(1) |
| N | 0.0040(5) | $C_2H_4$ ($\tau = 0$) | 0.011(3) | $C_2H_4$ ($\phi = 20$) | 0.002(2) | $C_2H_4$ ($\phi = 105$) | 0.007(2) |
| O | 0.0035(4) | $C_2H_4$ ($\tau = 15$) | 0.007(3) | $C_2H_4$ ($\phi = 40$) | 0.003(2) | $C_2H_4$ ($\phi = 110$) | 0.009(1) |
| LiH | 0.0047(9) | $C_2H_4$ ($\tau = 30$) | 0.004(3) | $C_2H_4$ ($\phi = 60$) | 0.001(1) | $C_2H_4$ ($\phi = 120$) | 0.002(3) |
| BeH | 0.0068(5) | $C_2H_4$ ($\tau = 45$) | 0.003(2) | $C_2H_4$ ($\phi = 70$) | 0.002(2) | $C_6H_6$ | 0.001(4) |
| Be (many states) | 0.0035(5) | $C_2H_4$ ($\tau = 60$) | 0.008(2) | $C_2H_4$ ($\phi = 80$) | 0.002(3) | | |
| BH | 0.0033(9) | $C_2H_4$ ($\tau = 70$) | 0.002(2) | $C_2H_4$ ($\phi = 90$) | 0.006(2) | | |

## SUPPLEMENTARY NOTE VII: HYPERPARAMETERS

We tabulate the hyperparameters that were used in all calculations. These are listed in Supplementary Table VII. We also tabulate the active spaces and spin configurations ($S^2$, $M = (N_\uparrow - N_\downarrow)/2$ (eigenvalue of $\mathcal{S}_z$)) used to generate the CASSCF baselines. These are listed in Supplementary Table VIII.

Supplementary Table VII: Hyperparameters used in calculations. (See Ref. [4] for more details.)

| Hyperparameter | Value | Hyperparameter | Value |
|---|---|---|---|
| One-electron basis | 6-311G | Maximum number of determinants | 10 |
| Dimension of $\mathbf{e}$ (# distance features) | 32 | Dimension of $\mathbf{x}_i$ (embedding dimension) | 128 |
| Dimension of $\mathbf{z}_i$ (kernel dimension) | 64 | Number of layers in $\mathbf{w}_{\boldsymbol{\theta}}$ | 1 |
| Number of layers in $\mathbf{h}_{\boldsymbol{\theta}}$ | 2 | Number of layers in $\mathbf{g}_{\boldsymbol{\theta}}$ | 2 |
| Number of interaction layers L: | | Number of layers in $\eta_{\boldsymbol{\theta}}$: | |
| small/intermediate systems & ethylene | 4 | small/intermediate systems & ethylene | 3 |
| benzene | 5 | benzene | 5 |
| Number of layers in $\kappa_{\boldsymbol{\theta}}$: | | Number of walkers: | |
| small/intermediate systems & ethylene | 3 | small/intermediate systems & ethylene | 1000 |
| benzene | 5 | benzene | 400 |
| Batch size | 2000 | Number of equilibration steps | 500 |
| Number of training steps: | | Optimizer | AdamW |
| small systems | 5000/10000 | Learning rate scheduler | CyclicLR |
| intermediate systems | 20000 | Minimum/maximum learning rate: | |
| ethylene | 10000 | small/intermediate systems & ethylene | 0.0001/0.005 |
| benzene | 10000 | benzene | 0.0001/0.001 |
| Cyclic frequency | 1000 | Clipping window q | 5 |
| Minimum/maximum $\alpha$ | 0.25/1.0 | Epoch size | 10 |
| Resampling frequency | 3 | Number of decorrelation sampling steps: | |
| Target acceptance | 57% | small/intermediate systems & ethylene | 5 |
| | | benzene | 15 |

Supplementary Table VIII: The active spaces, specifying $N$ electrons across $P$ orbitals, and spin configurations, used to generate the CASSCF baselines for all systems.

| System | CAS$(P, N)$ | Spin$(S^2, M)$ | System | CAS$(P, N)$ | Spin$(S^2, M)$ |
|---|---|---|---|---|---|
| Li | $(5, 3)$ | $(\frac{3}{4}/\frac{3}{4}/\frac{3}{4}/\frac{3}{4}/\frac{3}{4}, 1)$ | Be (many states) | $(6, 4)$ | (0/2/2/2/0/0/0/2, 1) |
| Be | $(5, 4)$ | (0/2/2/2/0, 0) | BH | $(5, 2)$ | (0/2/2/0, 0) |
| B | $(5, 5)$ | $(\frac{3}{4}/\frac{3}{4}/\frac{3}{4}/\frac{15}{4}/\frac{15}{4}, 1)$ | $CH^+$ | $(5, 2)$ | (0/2/2/0, 0) |
| C | $(5, 6)$ | (2/2/2/0/0, 0) | $H_2O$ | $(5, 2)$ | (0/2/0, 0) |
| N | $(5, 7)$ | $(\frac{15}{4}/\frac{3}{4}/\frac{3}{4}/\frac{3}{4}/\frac{3}{4}, 1)$ | $NH_3$ | $(7, 2)$ | (0/2/0, 0) |
| O | $(5, 8)$ | (2/2/2/0/0, 0) | CO | $(10, 10)$ | (0/2/0, 0) |
| LiH | $(12, 4)$ | (0/2/0/2/2/0/0/2, 0) | $C_2H_4$ | $(4, 4)$ | (0/0, 0) |
| BeH | $(12, 5)$ | $(\frac{3}{4}/\frac{3}{4}/\frac{3}{4}/\frac{3}{4}/\frac{15}{4}/\frac{15}{4}/\frac{3}{4}/\frac{3}{4}, 1)$ | $C_6H_6$ | $(4, 4)$ | (0/2, 0) |

## Supplementary References


[1] D. P. O'eill and P. M. W. G. *, Benchmark correlation energies for small molecules, Molecular Physics **103**, 763 (2005).

[2] E. Giner, D. Traore, B. Pradines, and J. Toulouse, Self-consistent density-based basis-set correction: How much do we lower total energies and improve dipole moments?, The Journal of Chemical Physics **155**, 044109 (2021).

[3] R. D. J. III, NIST Computational Chemistry Comparison and Benchmark Database, NIST Standard Reference Database Number 101 (2021).

[4] J. Hermann, Z. Schätzle, and F. Noé, Deep-neural-network solution of the electronic schrödinger equation, Nature Chemistry **12**, 891 (2020).
